# Presence of Tablet Remnants of Nevirapine Extended-Release in Stools and Its Impact on Virological Outcome in HIV-1-Infected Patients: A Prospective Cohort Study

**DOI:** 10.1371/journal.pone.0140574

**Published:** 2015-10-14

**Authors:** Yi-Chieh Lee, Shu-Wen Lin, Mao-Yuan Chen, Sui-Yuan Chang, Ching-Hua Kuo, Wang-Huei Sheng, Szu-Min Hsieh, Hsin-Yun Sun, Hsi-Yen Chang, Mon-Ro Wu, Wen-Chun Liu, Pei-Ying Wu, Shang-Ping Yang, Jun-Yu Zhang, Yi-Ching Su, Yi-Zhen Luo, Chien-Ching Hung, Shan-Chwen Chang

**Affiliations:** 1 Department of Internal Medicine, Lotung Poh-Ai Hospital, Lo-Hsu Foundation, Inc., I-Lan, Taiwan; 2 Department of Pharmacy, National Taiwan University Hospital and National Taiwan University College of Medicine, Taipei, Taiwan; 3 Graduate Institute of Clinical Pharmacy, National Taiwan University College of Medicine, Taipei, Taiwan; 4 Department of internal Medicine, National Taiwan University Hospital and National Taiwan University College of Medicine, Taipei, Taiwan; 5 Department of Laboratory Medicine, National Taiwan University Hospital and National Taiwan University College of Medicine, Taipei, Taiwan; 6 Department of Clinical Laboratory Sciences and Medical Biotechnology, National Taiwan University College of Medicine, Taipei, Taiwan; 7 School of Pharmacy, National Taiwan University, Taipei, Taiwan; 8 Center of Infection Control, National Taiwan University Hospital, Taipei, Taiwan; 9 Department of Medical Research, China Medical University Hospital, Taichung, Taiwan; 10 China Medical University, Taichung, Taiwan; University of British Columbia, CANADA

## Abstract

**Background:**

Nevirapine extended-release (NVP-XR) taken once daily remains an effective antiretroviral agent for patients infected with HIV-1 strains that do not harbor resistance mutations. Presence of tablet remnants of NVP XR in stools was reported in 1.19% and 3.05% of subjects in two clinical trials. However, the prevalence may have been underestimated because the information was retrospectively collected in the studies.

**Methods:**

Between April and December 2014, we prospectively inquired about the frequency of noticing tablet remnants of NVP XR in stools in HIV-1-infected patients who switched to antiretroviral regimens containing NVP XR plus 2 nucleos(*t*)ide reverse-transcriptase inhibitors. Patients were invited to participate in therapeutic drug monitoring of plasma concentrations of NVP 12 or 24 hours after taking the previous dose (C12 and C24, respectively) of NVP XR using high-performance liquid chromatography. The information on clinical characteristics, including plasma HIV RNA load and CD4 lymphocyte count, at baseline and during follow-up was recorded.

**Results:**

During the 9-month study period, 272 patients switched to NVP XR-based regimens and 60 (22.1%) noticed tablet remnants of NVP XR in stools, in whom 54.2% reported noticing the tablet remnants at least once weekly. Compared with patients who did not notice tablet remnants, those who noticed tablet remnants had a higher mean CD4 lymphocyte count (629 vs 495 cells/mm^3^, *P* = 0.0002) and a similar mean plasma HIV RNA load (1.57 vs 1.61 log_10_ copies/mL, *P* = 0.76) on switch. At about 12 and 24 weeks after switch, patients who noticed tablet remnants continued to have a similar mean plasma HIV RNA load (1.39 vs 1.43 log_10_ copies/mL, *P* = 0.43; and 1.30 vs 1.37 log_10_ copies/mL, *P* = 0.26, respectively), but had a lower median NVP C12 (3640 vs 4730 ng/mL, *P* = 0.06), and a similar median NVP C24 (3220 vs 3330 ng/ml, *P* = 0.95) when compared with those who did not notice tablet remnants.

**Conclusions:**

The presence of tablet remnants of NVP XR in stools is not uncommon in HIV-1-infected Taiwanese patients receiving NVP XR-based antiretroviral regimens, which does not have an adverse impact on the virological and immunological outcomes.

## Introduction

Nevirapine (NVP), a potent non-nucleoside reverse-transcriptase inhibitor (nNRTI), has been an important component of antiretroviral therapy for HIV-1 infection with high efficacy and lower rates of metabolic complications over the past two decades, although the adverse effects such as hepatotoxicity and hypersensitivity have precluded it from the preferred antiretroviral regimens [1–3]. The 2NN study demonstrated comparable virological response between patients treated with NVP immediate-release [NVP IR]) 200 mg twice daily and those treated with NVP IR 400 mg once daily [2], and both groups of subjects had similar exposure to NVP as indicated by 24 hours area-under-the-curve (AUC_24h_) [4].

To reduce the pill burden and enhance convenience and adherence, NVP IR was replaced by NVP extended-release (NVP XR) tablet that contains 400 mg of NVP in a single tablet to be taken once daily. The NVP XR tablet contains hydrophilic polymer hydroxypropyl methylcellulose, which provides extended, controlled release of NVP in the gastrointestinal tract. Although the NVP XR formulation had a lower bioavailability than NVP IR, trough plasma concentration with NVP XR was similar to that of NVP IR and no virological failure was reported in patients treated with NVP XR formulations [5].

In two clinical trials (VERxVE and TRANxITION), the NVP XR formulation has demonstrated non-inferiority of efficacy and similar safety to NVP IR formulation, although the exposure to NVP and the peak NVP concentration are lower in patients taking NVP XR than those taking NVP IR [6,7]. Presence of tablet remnants of NVP XR in stools has been reported to occur in 1.19% and 3.05% of the subjects in the two trials, respectively, by retrospective investigation [8]. However, such a prevalence may have been underestimated due to the fact that the information was obtained by self-reporting. Another post-marketing population-based cohort study with NVP XR tablets reported that 31 out of 536 (6%) patients had “whole” tablet in their stools, which was not associated with adverse outcomes [9].

In this prospective cohort study, we aimed to determine the prevalence of tablet remnants of NVP XR in stools and to evaluate its impact on virological and immunological outcomes in HIV-1-infected patients who switched to NVP XR-based antiretroviral regimens. Therapeutic drug monitoring of plasma NVP concentrations was performed in a subgroup of patients switching to NVP XR-based regimens.

## Methods

### Patients

**Fig 1:**
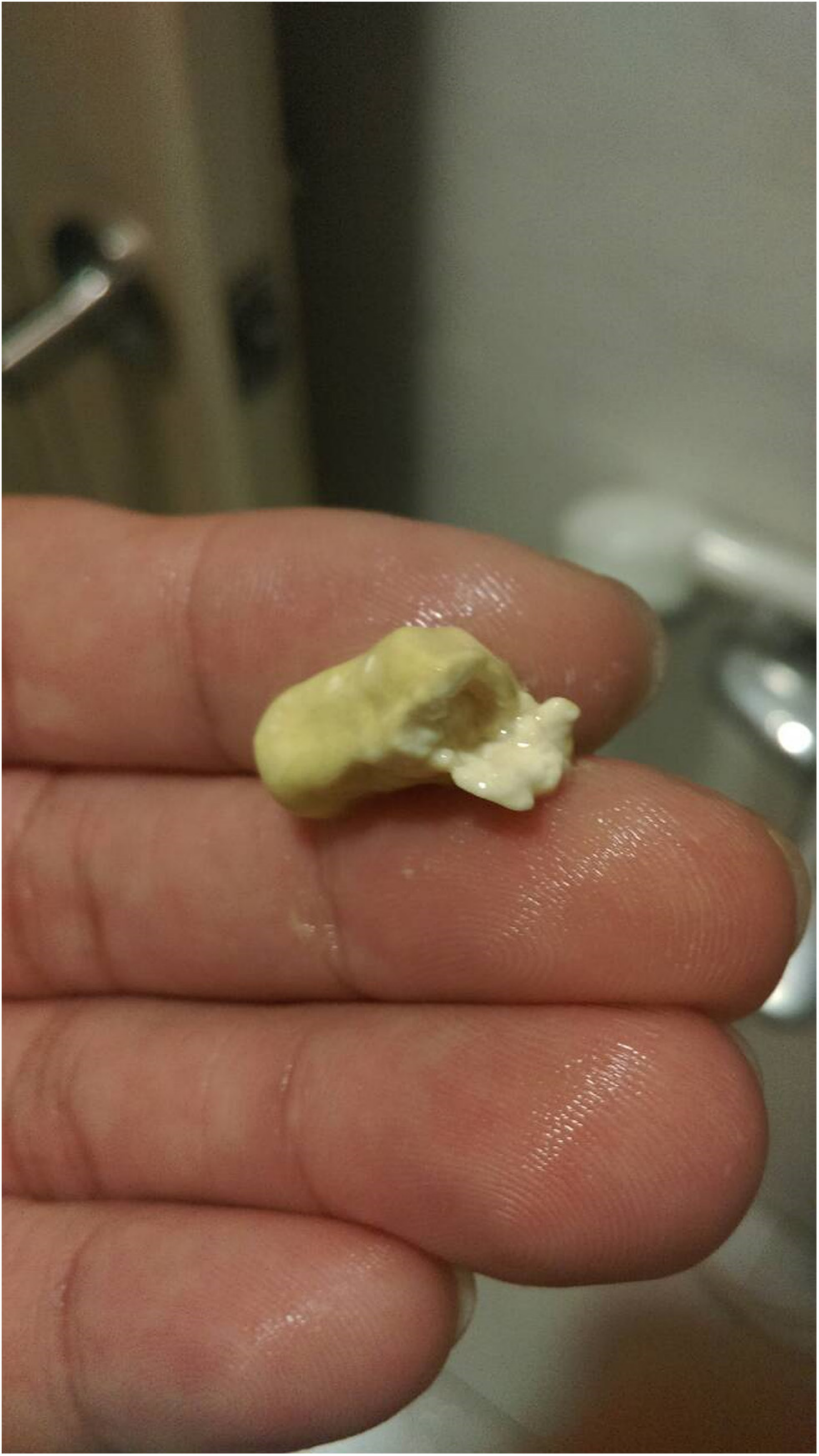
A photograph taken by one of the patients showing a washed tablet remnant of nevirapine extended-release collected from stools.

From April to December, 2014, we enrolled HIV-1-infected patients who switched to NVP XR plus 2 nucleos(*t*)ide reverse-transcriptase inhibitors. These patients were interviewed with the use of a face-to-face questionnaire to inquire about the frequency of noticing tablet remnants of NVP XR in stools (Fig 1) when the patients returned for the clinic appointments. Because the patients rarely paid attention to the contents of the stools, they were instructed to see if any tablet remnants were present in stools within the next 4 weeks after clinic visits and our case managers made cell phone calls to inquire about the results.

We used a computerized data collection form to record clinical characteristics that included age, gender, risk of HIV-1 transmission, co-infection of hepatitis B virus (HBV) or hepatitis C virus (HCV), frequency of noticing NVP XR remnants in stools, backbone antiretroviral therapy, duration of NVP XR before the study, concurrent medications, plasma HIV RNA load and CD4 lymphocyte count on and after switch to NVP XR-based regimens. Patients were invited to participate in therapeutic drug monitoring of plasma NVP concentrations with the use of high-performance of liquid chromatography (HPLC) 12 or 24 hours after taking the previous dose. Clinical characteristics, plasma NVP concentrations, and immunological and virological outcomes were compared between patients with and those without noticing tablets remnants of NVP XR in stools. The study was approved by the Research Ethics Committee of the hospital (registration numbers, 201103077RC and 20103112R) and the patients gave written informed consent before participating in the study.

### Laboratory investigations

After switch to NVP XR-based regimens, plasma HIV RNA load and CD4 lymphocyte count were followed every 3 to 6 months. CD4 lymphocyte count was determined using flow cytometry (BD FACS Calibur, Becton Dickinson and Coulter Epics XL, Beckman Coulter, CA, USA). Plasma HIV RNA load was quantified using the Cobas AmpliPrep/Cobas TaqMan HIV-I test (version 2.0, Roche Molecular Systems, Inc.) with a lower detection limit of 20 copies/mL.

Since 2009, we started to conduct therapeutic drug monitoring of plasma concentrations of efavirenz, nevirapine and atazanavir among HIV-infected patients [10]. Patients’ blood samples were collected into potassium and ethylenediaminetetraacetic-acid–containing 10-mL tubes. They were centrifuged and plasma was stored at -20°C prior to analysis. The plasma concentration of NVP was analyzed using HPLC with a modified method reported by van Heeswijk [11]. In brief, 300 μL of the plasma were mixed with 300 μL of acetonitrile for 30 seconds. The tubes were then centrifuged for 10 minutes at 10500 g, and 300 μL of the clear supernatant was evaporated to dryness under a gentle stream of nitrogen at 30°C. The residues were then dissolved in 200 μL of mobile phase, mixed for 30 seconds and centrifuged for 3 minutes at 10500 g. The clear supernatants were transferred to HPLC autosampler vials with inserts. The HPLC system consisted of a L-2130 HTA solvent delivery pump, a L-2200 autosampler, a L-2420 UV-Vis detector, and the HPLC D-2000 Elite on Windows (version 1.2, Hitachi High Technologies Corporation, Tokyo, Japan) chromatographic data system. Chromatography was performed on a Mightysil RP-18 GP column (250 x 4.6 nm, 5 μm; Kanto Corporation, Portland, OR). The mobile phase was composed of the phosphate buffer (pH = 4.0, 10 mM) mixed with acetonitrile at a ratio of 70:30 (vol/vol), and the flow rate was 1 mL/min. The detection wavelength was at 250 nm. The retention time of NEV was 6.31 minutes. The calibration curve of NVP was linear over the range 100 to 10000 ng/mL (y = 54403X+5045.3, R = 0.9997). The lower limit of quantification was 100 ng/mL. The extraction recovery was 97.22%. The accuracy ranged from 95.10 to 102.61%. The peak area of intra-assay and inter-assay coefficients of variation at 5000 ng/mL ranged from 0.09 to 0.58% and 0.12 to 0.63%, respectively.

### Statistical analysis

Statistical analyses were performed using SPSS software version 17.0 (SPSS Inc., Chicago, IL, USA). Continuous variables were reported as mean ± standard deviation, and compare with Student’s *t* test. Categorical variables were expressed as percentage of the total number of patient analyzed, and compare with chi-squared test. Plasma NVP concentrations of patients who noticed tablet remnants and those who did not were expressed by median and interquartile range (IQR) and were compared by Kruskal-Wallis test. A *P* value less than 0.05 was considered statistical significance.

## Results

During the 9-month study period, 310 patients who had switched to NVP XR plus 2 nucleos(*t*)ide reverse-transcriptase inhibitors were identified from the Department of Pharmacy of the hospital; 272 patients (87.7%) agreed to participate in the questionnaire interview, in whom 60 patients (22.1%) reported noticing tablet remnants of NVP XR in stools on face-to-face or cell phone questionnaire interview. The baseline clinical characteristics, virological and immunogical outcomes of HIV-1-infected patients with or without tablet remnants of NVP XR in stools are summarized in Table 1. Frequency of noticing NVP XR tablet remnants in stools ranged from once per day to 1 to 2 times every 3 months, with 53.3% of the patients noticing tablet remnants in stools at least once weekly (Table 2).

**Table 1:** Comparisons of baseline clinical characteristics of HIV-1-infected patients who noticed and those who did not notice tablet remnants of nevirapine extended-release (NVP-XR) in stools.

Variables	NVP XR remnant n = 60	No remnant n = 212	Statistics *P* value
Age, mean (SD), years	34.7 (8.0)	37.9 (11.9)	0.02
Male sex, n (%)	60 (100.0)	202 (95.3)	0.09
Risk for HIV transmission, n (%)			0.13
MSM/bisexual	58 (96.67)	189 (89.57)	
Heterosexual	1 (1.67)	20 (9.48)	
Others	1 (1.67)	3 (1.11)	
HBsAg-positive, n (%)	4 (6.67)	15 (7.08)	0.91
Anti-HCV-positive	2 (3.33)	17 (8.02)	0.21
Concurrent medications, n (%)			
Anti-hypertensives	2 (3.33)	14 (6.60)	0.34
Anti-diabetes	1 (1.67)	5 (2.36)	0.75
Lipid-lowering agents	1 (1.67)	10 (4.72)	0.29
Anti-HCV therapy	0	0	
PVL on switch to NVP XR, mean (SD), log_10_ copies/ml,	1.57 (0.84)	1.61 (0.94)	0.76
PVL on switch to NVP XR, PVL <50 copies/ml, n (%)	53 (88.33)	180 (86.12)	0.66
CD4 on switch to NVP XR, mean (SD), cells/mm^3^	629 (271)	495 (227)	0.0002
Backbone ARV, n (%)			
Abacavir/lamivudine	16 (26.67)	64 (30.19)	0.60
Tenofovir/lamivudine or tenofovir/emtricitabine	38 (63.33)	115 (54.25)	0.21
Zidovudine/lamivudine	4 (6.67)	32 (15.09)	0.09
Others	2 (3.33)	1 (0.47)	0.06
Duration of exposure to NVP XR before enrollment, mean (SD), days,	158 (66)	160 (73)	0.84

**Abbreviations:** ARV, antiretroviral therapy; HBsAg, hepatitis B surface antigen; HCV, hepatitis C virus; MSM, men who have sex with men; PVL, plasma HIV-1 RNA load; SD, standard deviation

**Table 2:** Frequency of noticing nevirapine extended-release (NVP-XR) tablet remnants and comparisons of virological and immunological outcomes of HIV-1-infected patients with and without noticing tablet remnants of NVP XR in stools.

Variables	NVP XR remnants n = 60	No remnants n = 212	Statistics *P* value
Frequency of NVP XR remnants in stools, n (%)			
Daily	6 (10.0)	NA	
2–5 times weekly	9 (15.0)	NA	
Once weekly	17 (28.3)	NA	
2–3 times monthly	6 (10.0)	NA	
Once monthly	12 (20.0)	NA	
1–2 times over a 3-month period	9 (15.0)	NA	
First PVL after switch to NVP XR, mean ± SD, log_10_ copies/ml, (n = 59/207)	1.39 (0.30)	1.43 (0.48)	0.43
First PVL < 50 copies/ml, n (%)	55 (93.2)	191 (92.3)	0.81
First CD4 after switch to NVP XR, mean (SD), cells/mm^3^, (n = 58/207)	660 (252)	541 (232)	0.0008
Interval between switch and first blood sampling, days, mean (SD)	95.6 (55.2)	101.5 (54.6)	0.47
Second PVL after switch to NVP XR, mean (SD), log_10_ copies/ml (n = 48/137)	1.3 (0.12)	1.37 (0.37)	0.26
Second PVL <50 copies/ml, n(%)	46 (96.0)	130 (95.0)	0.79
Second CD4 after switch to NVP XR, mean (SD), cells/mm^3^ (n = 48/134)	628 (270)	560 (229)	0.10
Interval between switch and second blood sampling, mean (SD), days	213 (72)	214 (58)	0.92

**Abbreviations:** ARV, antiretroviral therapy; MSM, men who have sex with men; NA, not applicable; PVL, plasma HIV-1 RNA load; SD, standard deviation; C12, plasma concentration 12 hours after the previous dosing; C24, plasma concentration 24 hours after the previous dosing

Compared with the patients who did not notice tablet remnants of NVP XR in stools, those who noticed tablet remnants were younger (34.7 vs 37.9 years, *P* = 0.02), and had similar mean plasma HIV RNA load (1.57 vs 1.61 log_10_ copies/mL, *P* = 0.76), a higher mean CD4 lymphocyte count (629 vs 495 cells/mm^3^, *P* = 0.0002) on switch to NVP XR-based regimens, and a similar duration of exposure to NVP XR before enrollment into this study (mean duration, 158 vs 160 days, *P* = 0.84) (Table 1).

After a mean duration of 100 days of switch to NVP XR in the first routine clinical follow-up, patients noticing tablet remnants had a similar mean plasma HIV RNA load (1.39 vs 1.43 log_10_ copies/mL, *P* = 0.43), a similar percentage of virological suppression (93.2% vs 92.3%, *P* = 0.81), and a higher mean CD4 lymphocyte count (660 vs 541 cells/mm^3^, *P* = 0.0008) compared with those who did not notice tablet remnants. Second clinical follow-up, at a mean duration of 213 days after switch, also demonstrated a similar mean plasma HIV RNA load (1.30 vs 1.37 log_10_ copies/mL, *P* = 0.26), percentage of virological suppression (<50 copies/ml) (96.0% vs 95.0%, *P* = 0.79), and mean CD4 lymphocyte count (628 vs 560 cells/mm^3^, *P* = 0.10) between the patients who noticed and those who did not notice tablet remnants (Table 2).

**Fig 2:**
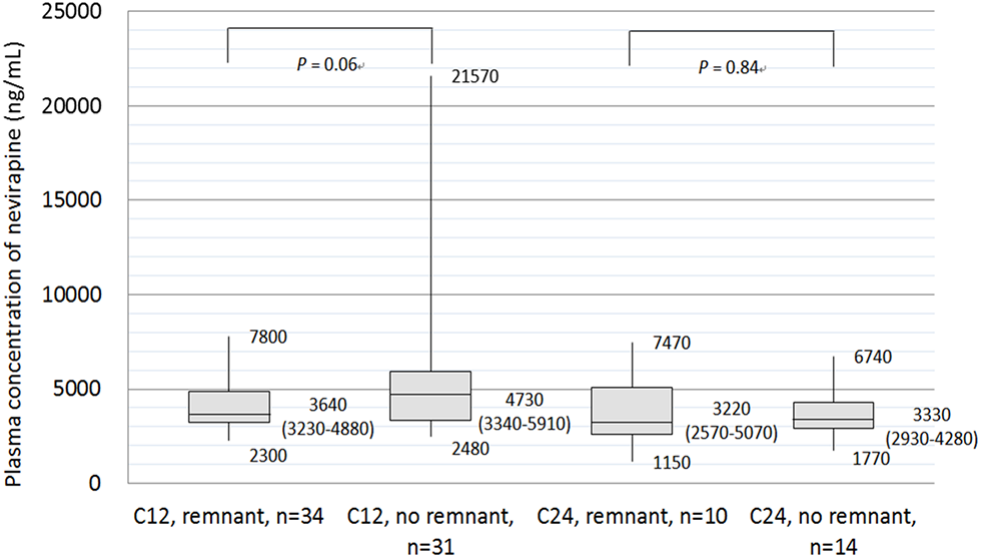
Plasma concentrations of nevirapine 12 hours (C12) and 24 hours (C24) following the previous dosing of nevirapine extended-release in patients noticing and those not noticing tablet remnants in stools. (Numbers in box plots indicate the median, interquartile range, maximum and minimum values).

A total of 89 patients (32.7%) participated in therapeutic drug monitoring of plasma NVP concentrations: 44 patients noticing tablet remnants (including 34 and 10 patients who had determinations of plasma NVP concentrations at 12 and 24 hours of the previous dosing, respectively) and 45 patients not noticing remnants (including 31 and 14 patients who had determinations of plasma NVP concentrations at 12 and 24 hours of the previous dosing, respectively). The clinical characteristics were similar between the two subgroups of patients who underwent therapeutic drug monitoring of plasma NVP concentrations (Table 3). The median plasma NVP concentration at 12 hours of the previous dosing was lower for the 34 patients who noticed tablet remnants in stools than that for the 31 patients who did not notice remnants (3640 ng/mL [IQR 3230–4880] vs 4730 ng/mL [IQR 3340–5910], *P* = 0.06), while the median plasma NVP concentrations at 24 hours of the previous dosing were similar between the two groups (3220 ng/mL [IQR 2580–5070] vs median 3330 ng/mL [IQR 2930–4280], *P* = 0.84) (Fig 2).

**Table 3:** Comparisons of clinical characteristics and virological and immunological outcomes of HIV-1-infected patients with and without noticing tablet remnants of nevirapine extended-release (NVP-XR) in stools, who underwent therapeutic drug monitoring of plasma concentration of nevirapine.

Variables	NVP XR remnants n = 44	No remnants n = 45	Statistics *P* value
Age, mean (SD), years	34.7 (7.82)	37.1 (11.06)	0.24
Male sex	44 (100.0)	43 (95.56)	0.16
Risk for HIV transmission			0.09
MSM/bisexual, n (%)	42 (95.45)	39 (86.67)	
Heterosexual, n (%)	1 (2.27)	6 (13.33)	
Others, n(%)	1 (2.27)	0 (0.00)	
HBsAg-positive, n (%)	2 (4.55)	1 (2.22)	0.54
Anti-HCV-positive, n (%)	0 (0.00)	2 (4.44)	0.16
Concurrent medications			
Anti-hypertensives	2 (4.55)	4 (8.89)	0.41
Anti-diabetes	1 (2.27)	1 (2.22)	0.99
Lipid-lowering agents	0 (0.00)	2 (4.44)	0.16
Anti-HCV therapy	NA	NA	NA
PVL on switch to NVP XR, mean (SD), log_10_ copies/ml	1.51 (0.66)	1.39(0.29)	0.33
CD4 on switch to NVP XR, mean (SD), cells/mm^3^	598.3 (249.8)	495.8 (201.1)	0.07
Backbone ARV, n (%)			
Abacavir/lamivudine	12 (27.3)	9 (20.0)	0.42
Tenofovir/lamivudine or tenofovir/emtricitabine	27 (61.4)	35 (77.8)	0.09
Zidovudine/lamivudine	3 (6.8)	1 (2.2)	0.29
Others	2 (4.6)	0 (0.0)	0.15
Duration of exposure to NVP XR before study, mean (SD), days	150 (65.7)	140 (57.7)	0.45
Frequency of NVP XR remnant in stools			
Daily	4 (9.09)	NA	
2–5 times weekly	9 (20.45)	NA	
Once weekly	12 (27.27)	NA	
2–3 times monthly	4 (9.09)	NA	
Once monthly	7 (15.91)	NA	
1–2 times over 3-month period	8 (18.18)	NA	
First PVL after switch to NVP XR, mean (SD), log_10_ copies/ml (n = 43/45)	1.38 (0.30)	1.41 (0.39)	0.70
First CD4 after switch to NVP XR, mean (SD), cells/mm^3^ (n = 42/44)	657 (265)	539 (226)	0.03
Interval between switch and first blood sampling, mean (SD), days	93 (52)	105 (62)	0.31
Second PVL after switch to NVP XR, mean (SD), log_10_ copies/ml (n = 37/30)	1.31 (0.06)	1.34 (0.10)	0.19
Second CD4 after switch to NVP XR, mean (SD), log_10_ copies/ml (n = 37/29)	629 (279)	562 (260)	0.32
Interval between switch and second blood sampling, mean (SD), days	213 (75.5)	212 (52.3)	0.99
C12 of NVP, median (IQR), ng/mL (n = 34/31)	3640 (2330–4880)	4730 (3340–5910)	0.06
C24 of NVP, median (IQR), ng/mL (n = 10/14)	3220 (2570–5070)	3330 (2930–4280)	0.95

**Abbreviations:** ARV, antiretroviral therapy; MSM, men who have sex with men; NVP XR, nevirapine extended-release; PVL, plasma HIV-1 RNA load; SD, standard deviation; C12, plasma concentration 12 hours after previous dosing; C24, plasma concentration 24 hours after previous dosing; IQR: interquartile range

## Discussion

This is the first prospective cohort study to determine the frequency of noticing tablet remnants of NVP XR in stools and its impact on virological and immunological outcomes in HIV-1- infected patients. Using a face-to-face and cell phone questionnaire interviews, we found that the prevalence of noticing tablet remnants of NVP XR in stools was significantly higher than those reported in two previous clinical trials (22.1% vs 1.19% and 3.05%, respectively) [6–8]; the prevalences of the two trials may have been underestimated because the information on noticing tablet remnants of NVP XR in stools was retrospectively obtained by self reporting of the patients. The higher prevalence of noticing tablet remnants of NVP XR in our study may be overestimated due to potential “questionnaire bias”, however. The way we obtained the information (face-to-face or cell phone questionnaire interview) might cause the patients to provide anticipated responses. On the contrary, we only performed the questionnaire interview once in a clinical visit or over the next 4 weeks of the visits; the cumulative rate of noticing NVP tablet remnants could be higher.

While the frequencies of tablet remnants of NVP XR in stools differ between our study and the study by Stephan and colleague [8], our findings support the conclusion made by Stephan and colleague that passing NVP XR tablet remnants in stools did not affect the treatment responses to NVP XR-based regimens in the two clinical trials because most of the active ingredient of NVP was absorbed with the residual NVP in the remnants ranging from 22.8 to 44.2% [8]. In our study, we did not observe any adverse impact on the immunological and virological responses to NVP XR-base regimens, either, given the findings that 22.1% of our patients noticed NVP XR remnants in stools and that the median plasma NVP concentration at 12 hours of the previous dosing tended to be lower in the patients with tablet remnants in stools than that in those without (3640 vs 4730 ng/mL, *P* = 0.06).

Therapeutic drug monitoring of NVP concentrations was recommended for efficacy and safety in patient care [12]. At a dose of NVP IR 200 mg twice daily, high exposure to NVP in plasma is associated with an improved virological response [13]. Minimum trough concentration of >3000 ng/mL for NVP had been suggested in a study that included patients with complete as well as those with incomplete adherence [14]. While the target trough concentration for other nevirapine formulations has not been established, Battegay and colleagues found that NVP XR provided a similar trough concentration as NVP IR [5]. In our patients with tablets remnants of NVP XR in stools, who may potentially have sub-therapeutic drug levels, their median trough concentration of NVP remained above the recommended therapeutic drug level (3220 ng/mL). This could explain why tablets remnants of NVP XR in stools had no adverse impact on virological outcomes in our study.

Passage of tablet remnants in stools is not an unusual phenomenon in clinical practices, which may occur whenever patients receive certain pharmaceutical agents that are designed to prolong the rate of drug release [15]. The active compound is continuously released during intestinal passage and its insoluble coats are visibly excreted into stools wholly or in part. The U.S. Food and Drug Administration (FDA) has warned the presence of tablet remnants in stools of some medications, including NVP XR [16], and declared “this will not affect the way your medicine works”. The studies by Stephan and ours should provide sufficient scientific evidence to support the declaration with regard to the impact of NVP XR on virological or immunological outcome.

Our study has several limitations. First, we did not request the patients to bring along the tablet remnants to the clinics for confirmation. Residues of other concurrent medications or undigested materials may have been mistakenly identified as remnants of NVP XR. Second, we were not able to identify the risk factors predisposing to passage of tablet remnants of NVP XR in stools. Passage of tablet remnants of NVP XR may be related to bowel movement, which may have wide day-to-day variation with dietary habits and other factors among individuals. Third, the number of our patients participating in therapeutic drug monitoring of plasma concentrations of NVP remains small and metabolism of nNRTIs may vary with genetic polymorphisms among different ethnicities [10,17,18]. Therefore, our findings among Taiwanese patients may not be generalizable to patients of other ethnicities. Last, we did not measure the residual NVP in the tablet remnants due to limited capacity of the facilities in the laboratory as what Stephan and colleague had done [8].

We conclude that the presence of tablet remnants in stools of NVP XR was not uncommon in HIV-1-infected Taiwanese patients, which did not have an adverse impact on immunological and virological outcome of the patients who switched from NVP IR to NVP XR.
